# The snake’s skin: fibroblastic sheath after veno-venous ECMO?

**DOI:** 10.1186/s44158-025-00240-3

**Published:** 2025-04-15

**Authors:** Francesco Alessandri, Antonella Tosi, Francesco Pugliese

**Affiliations:** 1 Intensive Care Unit, Department of General, Specialistic Surgery, Sapienza” University of Rome, 00161 Rome, Italy; 2 Azienda Ospedaliera-Universitaria Policlinico Umberto I, 00161 Rome, Italy

**Keywords:** ECMO, Thrombus, Complication, IVC

To the editor,

A 46-year-old male with severe acute hypoxic respiratory failure due to *Legionella pneumoniae* infection required VV ECMO support. Cannulation was performed percutaneously using a 23-Fr, 55-cm multistage drainage cannula (HLS, Getinge®) with bioline coating in the left femoral vein and a 20-Fr, 15-m return cannula (OptiSite, Edwards®) in the right jugular vein. A loading dose of unfractionated heparin was administered at the time of cannulation, followed by continuous intravenous infusion. Anticoagulation was monitored using the activated partial thromboplastin time ratio (APTTr), with a target range of 1.4 to 1.8. No risk factors for thrombophilia were identified, and no significant drainage insufficiency was observed.

The patient was successfully weaned from ECMO after 15 days of support. Following the removal of the femoral cannula, point-of-care ultrasound revealed a significant echogenic mass floating in the inferior vena cava (Fig. 1; see Supplementary video S1).

**Fig. 1:**
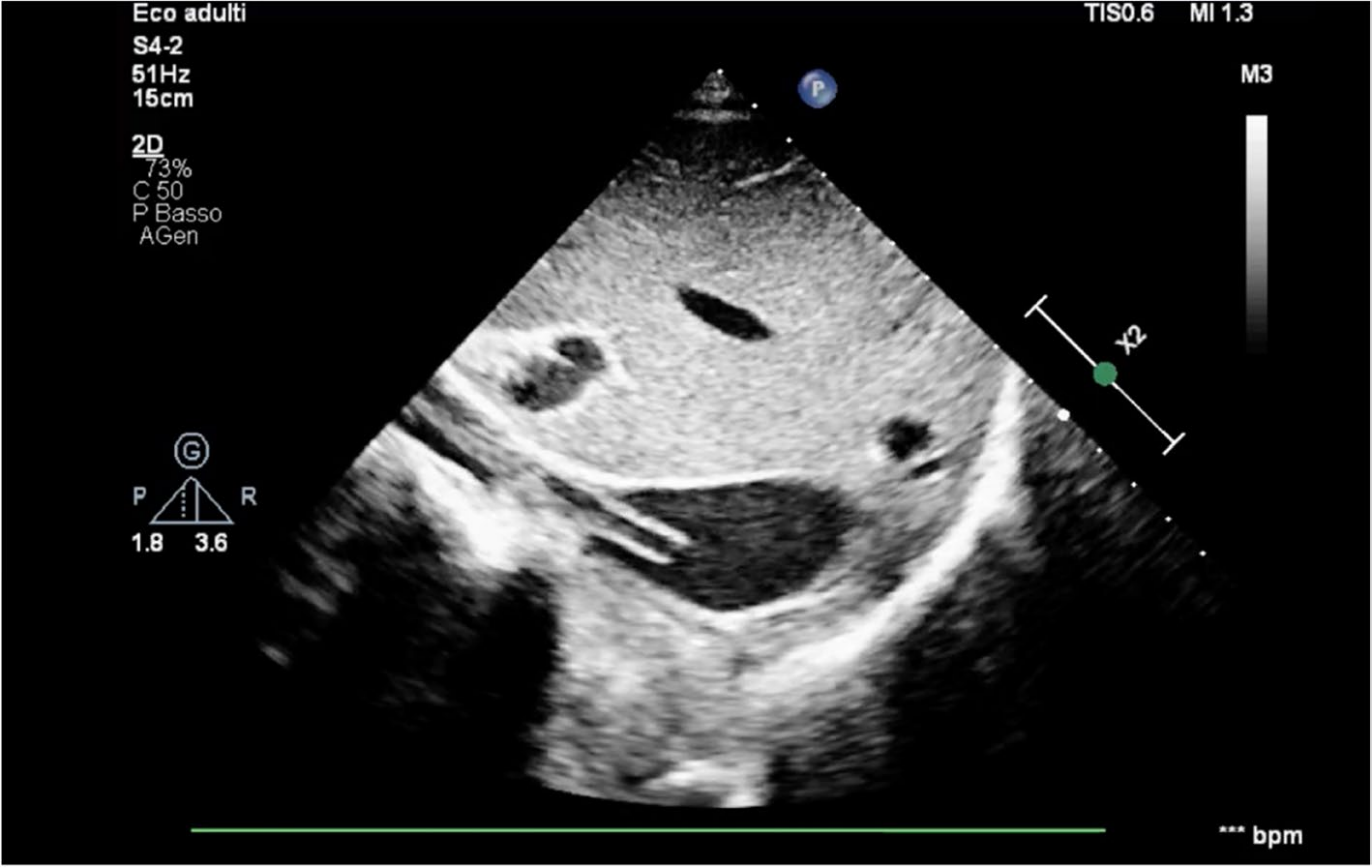
Ultrasound assessment of the inferior vena cava at its confluence with the right atrium: longitudinal view of a fibroblastic sheath

ECMO cannulas can generate echogenic sheaths that may attach to blood vessels and persist even after the cannula is removed; these may represent “fibroblastic sheaths”, whose primary non-cellular component consists of collagen, produced by fibroblasts, without fibrin [1, 2]. The formation of these fibroblastic sheaths is triggered by microtrauma to the endothelium and typically occurs within 24 h from the insult. Initially, a cellular tissue composed of smooth muscle cells develops at the cannula insertion site. After approximately 1 week, this cellular tissue evolves into a sheath made up of smooth muscle cells, collagen, and endothelial cells. After 2 weeks, the smooth muscle cells migrate from the vein wall to the sheath, forming cell bridges between the two structures.

Differentiating between ECMO-related venous thrombus and fibroblastic sheaths requires appropriate ultrasound training. This distinction is clinically significant, as a thrombus may necessitate anticoagulation treatment, while a fibroblastic sheath does not require pharmacological therapy. Fibroblastic sheaths develop around the catheter wall and can float freely in the venous vessel after cannula removal, often with little or no attachment to the vein wall. In contrast, a venous thrombus typically begins at the vein wall and exhibits significant adhesion to it. Additionally, venous thrombosis progresses gradually from an anechoic (fresh thrombus) to a hyperechoic (old thrombus) appearance, whereas the echogenic density of fibroblastic sheaths can be more difficult to predict. Most fibroblastic sheaths are easily identifiable because of their high echogenicity, although colour Doppler ultrasound may be necessary to detect hypoechoic sheaths. If these sheaths remain within the venous system, they could potentially dislodge and travel to the lungs or serve as a site for bacterial infection [3]. More commonly, the growth of fibroblastic sheaths on a multistage drainage cannula in the inferior vena cava can lead to refractory episodes of drainage insufficiency, increased resistance to inflow, or drainage failure due to increased negative pressure caused by unobstructed holes [4].

These findings highlight the importance of thorough monitoring of ECMO circuits and blood vessels for potential complications, as well as effective anticoagulation management throughout the procedure. During ECMO, repeated ultrasound assessments of the ECMO cannula’s wall thickness require substantial clinical expertise, and distinguishing between a fibroblastic sheath floating in the vessel after cannula removal and a hollow catheter-related thrombus can be challenging [5]. However, this differential diagnosis is critical in determining the appropriate treatment: thrombus presence warrants anticoagulation, whereas a fibroblastic sheath typically requires no treatment. Furthermore, anticoagulation protocols during veno-venous ECMO are based on expert consensus due to the limited research evidence. Consequently, investigating vascular complications associated with cannulation remains essential even after decannulation.

## Supplementary Information

Supplementary Material 1. Supplementary video S1. Point-of-care ultrasound revealed a significant echogenic mass floating in the inferior vena cava.

## Data Availability

No datasets were generated or analysed during the current study.
